# LTX-315 triggers anticancer immunity by inducing MyD88-dependent maturation of dendritic cells

**DOI:** 10.3389/fimmu.2024.1332922

**Published:** 2024-03-13

**Authors:** Xiao-Qing Li, Takahiro Yamazaki, Tianzhen He, Md Masud Alam, Jia Liu, Anna L. Trivett, Baldur Sveinbjørnsson, Øystein Rekdal, Lorenzo Galluzzi, Joost J. Oppenheim, De Yang

**Affiliations:** ^1^ Department of Biochemistry and Molecular Biology, Tianjin Medical University Cancer Institute and Hospital, National Clinical Research Center of Cancer, Tianjin Medical University, Tianjin, China; ^2^ Laboratory of Cancer Innovation, Frederick National Laboratory for Cancer Research, Center for Cancer Research, National Cancer Institute, Frederick, MD, United States; ^3^ Department of Radiation Oncology, Weill Cornell Medical College, New York, NY, United States; ^4^ Lytix Biopharma, Oslo, Norway; ^5^ Sandra and Edward Meyer Cancer Center, New York, NY, United States; ^6^ Caryl and Israel Englander Institute for Precision Medicine, New York, NY, United States

**Keywords:** antitumor immunity, dendritic cells, LTX-315, toll-like receptors, MyD88, melanoma

## Abstract

LTX-315 is a synthetic cationic oncolytic peptide with potent anticancer activity but limited toxicity for non-malignant cells. LTX-315 induces both immunogenic tumor cell death and generation of tumor-specific immune responses in multiple experimental tumor models. Given the central role of dendritic cell (DC) maturation in the induction of antigen-specific immunity, we investigated the effect of LTX-315 treatment on the maturation of tumor-infiltrating DCs (TiDCs) and the generation of anti-melanoma immunity. We found that LTX-315 treatment induces the maturation of DCs, both indirectly through the release of cancer cell-derived damage-associated molecular patterns (DAMPs)/alarmins and nucleic acids (DNA and RNA) capable of triggering distinct Toll-like receptor (TLR) signaling, and, directly by activating TLR7. The latter results in the ignition of multiple intracellular signaling pathways that promotes DC maturation, including NF-κB, mitogen activated protein kinases (MAPKs), and inflammasome signaling, as well as increased type 1 interferon production. Critically, the effects of LTX-315 on DCs the consequent promotion of anti-melanoma immunity depend on the cytosolic signal transducer myeloid differentiation response gene 88 (MyD88). These results cast light on the mechanisms by which LTX-315 induces DC maturation and hence elicits anticancer immunity, with important implications for the use of LTX-315 as an anticancer immunotherapeutic.

## Graphical Abstract

Schematic illustration of the direct and indirect DC-maturing effects of LTX- 315 and the sequential immune-mediated anti-cancer effects.

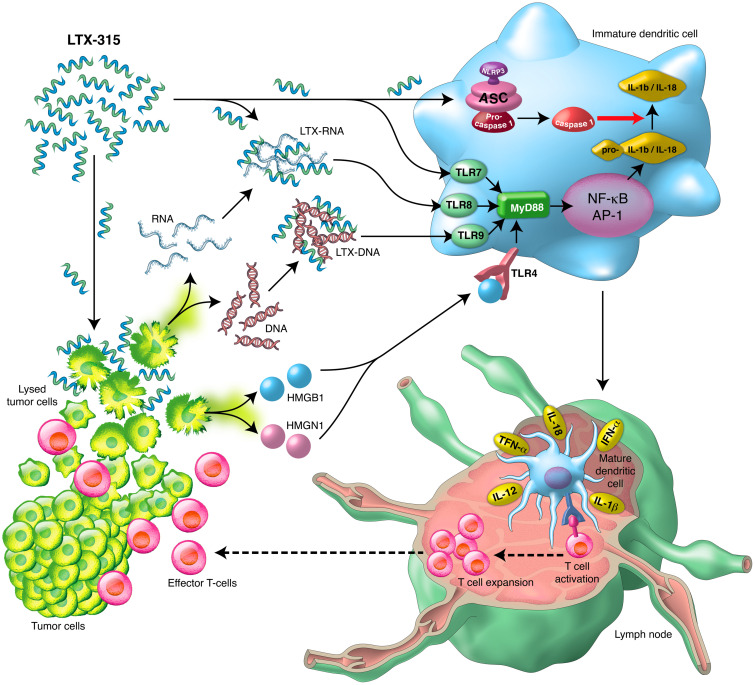

## Introduction

Cationic antimicrobial peptides (CAPs) are naturally occurring small peptide antibiotics that are integral components of the innate immune systems against microbial infection (1, 2). Common to all CAPs is their amphipathic nature, which enables them to integrate into anionic membrane lipid bilayers resulting in membrane disruption and ultimately permeabilization. In addition to their antimicrobial properties, many CAPs also display membranolytic activity on mammalian cells (1, 3). Due to a higher abundance of anionic membrane components than normal cells, cancer cells are generally more sensitive to the cytolytic activity of CAPs than their normal counterparts, which makes CAPs attractive candidates for the design and development of novel oncolytic anticancer peptides (3, 4).

LTX-315 is a synthetic 9-mer cationic oncolytic peptide developed as an analogue of bovine lactoferricin (5, 6), which was selected from a series of chemically modified lactoferricin-derived lytic peptides because it displayed superior anticancer activity and lower toxicity on normal cells (5). LTX-315 induces tumor cell death by rapidly damaging cell membrane integrity (6, 7) and permeabilizing mitochondrial membrane (8, 9). Intra-tumoral administration of LTX-315 stimulates the generation of systemic tumor-specific immune responses (10, 11), resulting in increased infiltration of cytotoxic CD8^+^ T cells and decreased regulatory T (Treg) cell infiltration in primary treated tumors (11–14) and in re-challenged secondary tumors (10). Delivery of LTX-315 into one tumor causes the regression of distal non-treated lesions (10) and the cured mice are protected against a re-challenge with the same cancer cells (10, 11). A phase I clinical study showed that LTX-315 converts immunogenically “cold” tumors to “hot” in patients with advanced or metastatic tumors (melanoma, sarcoma, or breast cancer), with increases in CD8^+^ tumor-infiltrating lymphocytes (TILs) in more than 80% of the patients and regression of distant tumor in some individuals (15). LTX-315 is currently under evaluation in a phase II clinical trial for the treatment of transdermally accessible cancers such as melanoma, lymphoma, soft tissue sarcoma, and squamous cell carcinoma (16, 17).

Beside inducing an immunogenic cell death (ICD), the mechanism by which LTX-315 treatment stimulates systemic tumor-specific immune responses is not fully educidated. In tumor-bearing hosts, the generation of tumor-specific immune responses requires effective presentation of tumor antigen(s) to T cells in the draining lymph nodes (dLNs) by antigen-presenting cells (APCs) migrating from malignant tissues. DCs are the main type of APCs that initiate and control the induction of adaptive (including antitumor) immune responses (18). For tumor-infiltrating DCs (TiDCs) to traffic to dLNs for the induction of antitumor immune responses, they must mature and acquire the necessary features capable of sufficiently triggering the activation of specific T cells. Given the release of multiple damage-associated molecular patterns (DAMPs) and alarmins (ATP, HMGB1, etc) by tumor cells treated with LTX-315 *in vitro* (8, 10, 11) and the known capacities of DAMPs/alarmins to induce DC maturation (19, 20), it has been proposed that the DAMPs/alarmins released by LTX-315-treated tumor cells are responsible for triggering the maturation of TiDCs and subsequent tumor-specific immune response (6). We therefore sought to investigate whether and how LTX-315 induces DC maturation in the context of the generation of anti-melanoma immunity. The results revealed that LTX-315 induced DC maturation *in vivo* and *in vitro*. In this context, we identified two additional pathways by which LTX-315 treatment triggered DC maturation: one involving direct activation of DCs via NF-κB, MAPKs, and inflammasome, and the other involving the formation of DC-maturing complexes between LTX-315 and DNA/RNA fragments released by LTX-315-treated melanoma cells. Importantly, LTX-315-induced TiDC maturation and the generation of anti-melanoma immunity relied on the presence of the signal transducer MyD88. Thus, LTX-315 triggers the generation of anti-melanoma immunity by inducing MyD88-dependent maturation of TiDCs.

## Materials and methods

### Reagents

LTX-315 (K-K-W-W-K-K-W-Dip-K-NH2) was produced and purchased on request from Bachem AG (Bubendorf, Switzerland) and was provided as a lyophilized sterile powder.

### Mice

WT C57BL/6, congenic TLR7^-/-^ and TLR9^-/-^ mice were provided by the animal production facility of the National Cancer Institute (NCI, USA). WT C57BL/6J and congenic MyD88^-/-^ mice were purchased from Jackson Laboratory (Bar Harbor, ME, USA). Female mice of 10- to 12-week-old were used to isolate mouse bone marrow mononuclear cells. Female mice of 7- to 8-week-old were used for B16F10 melanoma implantation. All mice were kept under specific pathogen-free conditions. All experiments with mice were performed in compliance with the principles and procedures outlined in the National Institutes of Health Guide for the Care and Use of Animals and were approved by the NCI Animal Care and Use Committee.

### Cell lines

Human melanoma A375, mouse melanoma B16F10, and mouse lymphoma EG7 cell lines were originally obtained from the American Type Culture Collection (ATCC, Manassas, VA, USA). A375 and B16F10 cells were maintained in DMEM medium (HyClone, Logan, UT, USA), and EG7 cells were maintained in RPIM 1640 medium (HyClone).

### Preparation and treatment of human and mouse DCs

Human peripheral blood was obtained from healthy donors with an approved human subject agreement. Human peripheral blood mononuclear cells (PBMCs) were isolated with 90-95% purity as previously described (21, 22). CD14^+^ monocytes were isolated from PBMCs using CD14^+^ Monocyte Isolation kit (Miltenyi Biotec, Auburn, CA, USA). Human monocyte-derived dendritic cells (monoDCs) were generated by culturing purified CD14+ monocytes in complete RPMI1640 medium containing 50 ng/ml of human GM-CSF and 50 ng/ml of human IL-4 in a CO2 incubator for 7 days. Human pDCs were isolated from human PBMCs using Plasmacytoid Dendritic Cell Isolation kit (Miltenyi Biotec) and immediately used in experiments. Mouse DCs (muDCs) were generated by culturing mouse hematopoietic progenitors (isolated from the femurs and tibias of c57BL/6 mice) in complete RPMI 1640 containing 20 ng/ml mouse GM-CSF for 6 days as previously described (21–23). Human and mouse DCs were treated with the indicated concentrations of activators for indicated periods of time before being analyzed for phenotype, function, or signaling. Lipopolysaccharide (LPS, 100 ng/ml), CpG-ODN (100 ng/ml) and R848 (10 ng/ml) were used as positive controls.

### siRNA and transfection

Human TLR8 siRNAs were purchased from Horizon Discovery LTD (Waterbeach, Cambridge, UK). TLR8 siRNA-1 and siRNA-2 target GAACGGAAAUCCCGGUAUA and CAGAAUAGCAGGCGUAACA, respectively. siRNA and control were transfected into monoDCs using Lipofectamine 3000 (Thermo Fisher Scientific, Waltham, MA, USA) following the vendor’s recommendation.

### *In vitro* cytotoxicity

For adherent A375 and B16F10, when the cells grown to 80% confluency in 24-well plate, cells were treated with various concentrations of LTX-315 for indicated time in FBS-free DMEM medium. For human monoDCs or muDCs, 1 x 10^6^ cells were seeded into 24-well plate and treated with various concentration of LTX-315 for specified time in FBS-free RPMI 1640 medium. Subsequently, treated melanoma cells and DCs were double stained with Annexin V-FITC and propidium iodide (PI) for flow cytometry analysis (Thermo Fisher Scientific).

### LTX-315 and nucleic acid complex formation

DNA and RNA were extracted from the supernatants of A375 and B16F10 cultures using QIAamp DNA Mini Kit (Qiagen, Germantown, MD, USA) and QIAamp RNeasy Kit (Qiagen) according to the vendor’s protocols. Indicated amounts of LTX-315 and DNA/RNA were diluted into 25 µl PBS respectively and subsequently mixed together for 30 min to form LTX-315-DNA or LTX-315-RNA complexes. The complexes were added into DC cultures or were observed under a fluorescence microscope for photography after staining with 1 µg/ml of propidium iodide (PI).

### Proteinase K, DNase I, and RNase A treatment

Proteinase K (100 µg/ml), DNase I (500 µg/ml) and RNase A (50 µg/ml) were used to digest the LTX-315-treated melanoma cell supernatant and LTX-315-nucleic acid complexes at 37°C for indicated time.

### Assessment of DC phenotype *in vitro*

The expression of surface molecules was determined by flow cytometry using a BD LRS II (BD Pharmingen, Franklin Lakes, NJ, USA) after staining with monoclonal antibodies against human CD80 (APC conjugated, BD Pharmingen), CD83 (PE conjugated, BD Pharmingen), CD86 (FITC conjugated, Biolegend, San Diego, CA, USA), HLA-DR (PB conjugated, Biolegend) or monoclonal antibodies against mouse CD11c (PE-Cy7 conjugated, Biolegend), CD80 (PE conjugated, Biolegend), CD86 (FITC conjugated, BD Pharmingen) and I-A/E (APC, BD Pharmingen), or isotype-matched control antibodies.

Intake of PI-labeled LTX-315-nucleic acid complex by monoDCs was observed under a fluorescence microscope and determined by flow cytometry. In brief, PI-labeled LTX-315-DNA and LTX-315-RNA complexes were washed twice to remove residual free PI before adding into DC cultures. Two hours later, DCs were analyzed by fluorescence microscopy or flow cytometry.

IL-1β, TNFα, and IL-12 in the supernatants of treated DCs were measured using V-PLEX ultrasensitive plate assay (Meso Scale Discovery, Rockville, MD, USA). IFNα was quantitated using ELISA kits (Thermo Fisher Scientific).

### Assessment of activation of NF-κB and MAPK signaling pathway

Human monoDCs were starved in FBS-free RPMI 1640 medium for overnight, followed by treatment with 20 ng/ml LTX-315 in complete RPMI 1640 for indicated periods of time. Treated DCs were lysed using RIPA (radio immunoprecipitation assay) lysis buffer. The DC lysate was collected and used to detect markers for NF-κB and MAPK signaling pathway by western blot.

### DC inflammasome activation

Human monoDCs were primed with 100 ng/ml LPS for 6 hours, and subsequently were stimulated with 20 μg/ml LTX-315 for 5 hours or with 5 mM ATP (Sigma-Aldrich, St. Louis, MO, USA) for 1 hour. Untreated samples, LTX-315 only treated samples, and LPS only treated samples were included as control for all experiments. Caspase 1 activity was determined using Caspase-Glo 1 Inflammasome Assay Kit (Promega, Madison, WI, USA). The levels of pro-caspase 1, pro-IL-1β, cleaved caspase 1 and cleaved IL-1β in cell lysate and supernatant were detected by western blot.

### Real-time RT-qPCR

Reverse transcription was performed using RT^2^ First Strand Kit (Qiagen) following the Vendor’s guideline. Quantitative Real-time PCR was utilized to detect the mRNA expression of *IFNA1* using a SYBR Green real-time PCR kit (Qiagen) and RT qPCR Primer Kits for *IFNA1* and *ACTB* (Qiagen). Assays were performed with the ABI 7500 TaqMan system (Applied Biosystems, Foster City, CA, USA).

### Western blot

Western blot was performed using the following primary antibodies: rabbit anti-HMGN1 (PreteinTech, Rosemont, IL, USA), rabbit anti-HMGB1 (Cell Signaling, Danvers, MA, USA), rabbit anti-phosphorylated p65 (Cell Signaling), rabbit anti-p65 (Cell Signaling), rabbit anti-IκBα (Cell Signaling), rabbit anti-phosphorylated p44/p42 (p-ERK ½, Cell Signaling), rabbit anti-p44/p42 (ERK ½, Cell Signaling Technology), rabbit anti-phosphorylated p38 (Cell Signaling), rabbit anti- p38 (Cell Signaling), mouse anti-phosphorylated JNK (Cell Signaling), rabbit anti-JNK (Cell Signaling), rabbit anti-pro-IL-1β (Novus Biologicals, Centennial, CO, USA), rabbit anti-IL-1β (Cell Signaling), rabbit anti-caspase 1 (Cell Signaling), mouse anti-TLR8 (Novus Biologicals) and rabbit anti-GAPDH (Cell Signaling).

### Mouse B16F10 melanoma model and treatment

C57BL/6J or congenic MyD88^-/-^ mice were subcutaneously inoculated into the right flank with 1.5 x 10^5^ mouse melanoma B16F10 cells to allow the formation of melanoma. To measure the maturation of TiDCs, B16-bearing mice with a size of 0.4-0.5 cm in diameter were intratumorally injected with 1 µg of FITC-OVA mixed with 1 mg of LTX-315 in 50 µl of PPBS or equal volume of PBS containing 1 µg FITC-OVA alone as negative control. After 48 h, mice were sacrificed for collecting tumor tissues and dLNs. Tumor tissues were minced and digested twice in dissociation solution (Leibovits L-15 containing 0.17 mg/ml collagenase I, 0.056 mg/ml collagenase II, 0.17 mg/ml collagenase IV, 0.025 mg/ml DNase I and 0.025 mg/ml elastase) at 37°C for 45 min to make single cell suspension. dLNs were digested once using the same dissociation solution. The percentages of various types of DCs in tumors and dLNs were determined by flow cytometry after staining with monoclonal antibodies against mouse CD45 (PB conjugated, Thermo Fisher Scientific), CD11c (PE conjugated, Biolegend) and B220 (APC conjugated, BD Pharmingen).

For therapeutic treatment, B16-bearing mice with a size of 0.2-0.3 cm in diameter were intratumorally injected with 3 doses (1 mg/50 µl PBS/injection) of LTX-315 on consecutive days, and the tumor growth was monitored. Tumor-bearing-mice were euthanized when the tumor reached 2 cm in diameter or if the mouse exhibited 20% weight loss or appeared obviously stressed. The tumor-free mice that resulted from LTX-315 treatment were re-challenged with either B16F10 (1.5 x 10^5^/inoculation) and EG7 (4 x 10^5^/inoculation) on contralateral flanks followed by monitoring of tumor appearance and growth for up to 3 weeks.

### Statistical analysis

All experiments were repeated two to three times. All quantified data are presented as mean ± standard deviation (SD). Kaplan-Meier Survival curves and the log-rank test were used to evaluate the survival status in mice experiments. Repeated measures analysis of variance (ANOVA) was used to compare the differences in B16 melanoma growth. All other comparisons were performed using two-tailed Student t-test or one-way ANOVA. All statistical tests were performed using GraphPad Prism software (GraphPad Software, Inc., San Diega, CA, USA).

## Results

### Intratumoral administration of LTX-315 triggers TiDC maturation

**Figure 1:**
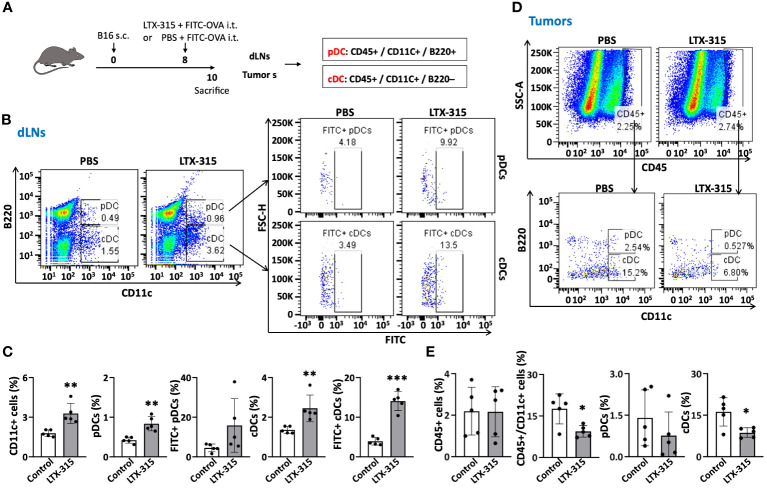
LTX-315 treatment triggers migration of TiDCs from tumor tissues to dLNs. **(A)** Schematic illustration of the experiments. C57BL/6J mice were subcutaneously (s.c.) injected into the right flank with 1.5 x 10^5^ B16F10 cells on day 0. On day 8, the B16-bearing mice were intratumorally (i.t.) injected with a mixture of FITC-OVA (1 µg) and LTX-315 (1 mg) or equal volume of PBS containing 1 µg FITC-OVA alone. Tumors and corresponding dLNs were collected and analyzed on day 10. **(B–E)**, Flow cytometry analysis of CD45^+^ leukocytes and various types of DCs (CD11c^+^ DCs, pDCs, cDCs, FITC^+^ pDCs and FITC^+^ cDCs) in dLNs and tumors. Shown are the representative plots **(B, D)** and average (mean ± SD, n=5) percentages **(C, E)** of dLN **(B, C)** and tumors **(D, E)**. *p < 0.05, **p < 0.01, ***p < 0.001 by Student’s t test in comparison with the control.

TiDCs need to mature to acquire the capacity to migrate to the dLNs to induce antitumor immune responses. Two major DC subsets infiltrate tumors: CD11C^+^/B220^+^ plasmacytoid DCs (pDCs) and CD11C^+^/B220^-^ conventional DCs (cDCs) (18, 24, 25). pDCs mainly produce type I interferons (IFNs) upon sensing prokaryotic DNA and CpG oligonucleotide. Upon maturation, cDCs migrate to dLNs to present tumor antigen(s) displayed on their surface in the context of MHC complexes to naïve T cells bearing TCR(s) specific for such antigen(s). To determine whether LTX-315 could trigger TiDC maturation, B16F10-bearing mice were injected intratumorally with LTX-315 and FITC-OVA (as a fluorescence tracer not affecting TiDC maturation) and their tumors and dLNs were resected 48 h later to evaluate TiDC migration to dLNs (**Figure 1A**). Both dLNs and tumors were dissociated into single cell suspensions using an enzymatic cocktail as previous reported (26), followed by immunostaining and analyzed by flow cytometry to quantitate the percentages of FITC-positive CD11C^+^ total DCs, CD11C^+^/B220^+^ pDCs and CD11C^+^/B220^-^ cDCs in dLNs and tumor tissues. The total DCs, pDCs and cDCs in dLNs of LTX-315-treated mice were dramatically increased comparing with those in sham-treated control mice (**Figures 1B, C**). Moreover, both FITC^+^ pDCs and FITC^+^ cDCs were also significantly increased in dLNs of LTX-315-treated mice (**Figures 1B, C**). When CD45^+^ tumor-infiltrating leukocytes were analyzed, the levels of CD45^+^ cells in tumor tissues were not significantly different between LTX-315 treated and control mice (**Figures 1D, E**). In contrast, CD45^+^/CD11c^+^ total DCs and CD45^+^/CD11c^+^/B220^-^ cDCs were markedly decreased in tumor tissues of LTX-315 treated mice compared to control mice (**Figures 1D, E**). These results indicated that intratumoral injection of LTX-315 triggers *in vivo* maturation of cDCs and pDCs, resulting in the migration TiDCs from tumor tissues to the dLNs.

### LTX-315-treated melanoma cells release molecules that promote DC maturation

**Figure 2:**
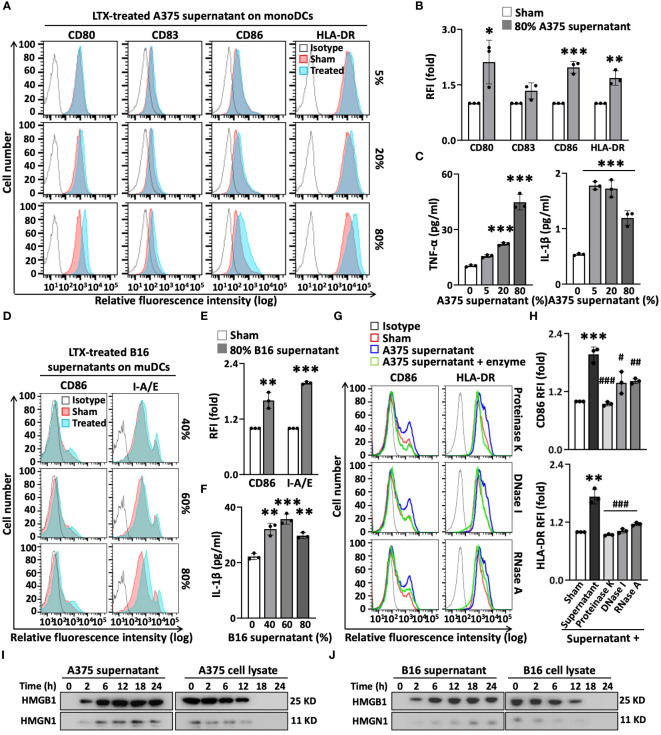
The supernatants of LTX-315(LTX)-treated melanoma cells contain elements capable of inducing DC maturation. The supernatants of melanoma (A375 or B16F10) cells were collected after treatment with LTX-315 (50 µg/ml) for 6 h. DCs incubated at 5 x 10^5^/ml in the absence (sham) or presence of indicated concentrations of tumor cell-derived supernatants for 48 h were analyzed by flow cytometry while their supernatants were used for the measurement of cytokines. **(A–F)**, human monoDCs **(A–C)** and mouse DCs **(D–F)** upregulated their expression of surface costimulatory (CD80, CD83, CD86) and MHC (HLA-DR, I-A/E) molecules **(A, B, D, E)** as well as the production of TNFα and/or IL-1β **(C, F)** in response to the supernatants of LTX-315-treated melanoma cells. **(G, H)**, A375-derived supernatants were digested at 37°C with proteinase K (100 µg/ml, 1 h), DNase I (500 µg/ml, 4 h) or RNase A (50 µg/ml, 4 h) before adding to DC cultures. Shown are the plots histograms of one experiment **(A, D, G)** and the average (mean ± SD) relative fluorescent intensity (RFI) or cytokine concentration of three independent experiments **(B, C, E, F, H)**. *p < 0.05, **p < 0.01, ***p < 0.001 by Student’s t-test in comparison with sham; ^#^ *p* < 0.05, ^##^ *p* < 0.01, ^###^ *p* < 0.001 by One-way ANOVA in comparison with native supernatant. **(I-J)**, Western blot detection of HMGB1 and HMGN1 in the supernatants and cell lysates of A375 **(I)** or B16 **(J)** melanoma cells after treatment with LTX- 315 (50 µg/ml) for the time indicated.

Although it was proposed that DAMPs/alarmins released by necrotic tumors resulting from LTX-315 treatment are perhaps responsible for inducing the maturation of DCs (6), this has not been experimentally demonstrated. LTX-315 induced necrotic death of both A375 and B16F10 tumor cells in a dose- and time-dependent manner, as shown by microscopy and staining with annexin V and propidium iodide (PI) (**Supplementary Figures 1A–D**). LTX-315 mediated significantly lower toxicity on DCs compared to tumor cells (**Supplementary Figures 1E, F**). To determine the mechanism(s) by which LTX-315 treatment causes DC maturation, we first investigated whether the supernatants of A375 and B16F10 melanoma cells treated with LTX-315 would induce the maturation of human and mouse DCs, respectively. Indeed, the supernatant of LTX-315-treated A375 cells significantly upregulated the expression of co-stimulatory (CD80, CD83, CD86) and MHC class II (HLA-DR) molecules on the surface of human monocyte-derived DCs (monoDCs) in a dose-dependent manner (**Figures 2A, B**). Furthermore, the supernatant of LTX-315-treated A375 cells also stimulated the production of pro-inflammatory cytokines tumor necrosis factor alpha (TNF-α) and interleukin 1beta (IL-1β) by human monoDCs (**Figure 2C**). Similarly, the supernatant of LTX-315-treated B16F10 cells promoted the maturation of mouse DCs, as illustrated by the upregulation of surface expression of CD86 and I-A/E (**Figures 2D, E**), and the production of pro-inflammatory cytokine IL-1β (**Figure 2F**). Importantly, the supernatants of A375 and B16F10 cells cultured in the absence of LTX-315 did not induce the maturation of human and mouse DCs, respectively (**Supplementary Figures 2A-D**). Therefore, LTX-315-treated melanoma cells release component(s) that can induce DC maturation.

To identify such components, we treated the supernatant of LTX-315-treated A375 cells with proteinase K, DNase I, or RNase A, and subsequently used it to stimulate human monoDCs. The capacity of the supernatant of LTX-315-treated A375 cells to upregulate the expression of surface CD86 and HLA-DR on DC surface was diminished to various degree by digestion with proteinase K, DNase I, or RNase A (**Figures 2G, H**). Of note, an identical amount of just proteinase A, DNase I, or RNase A alone did not affect the expression levels of CD86 and HLA-DR on DC surface (**Supplementary Figure 3**), ruling out the possibility that the enzyme preparation somehow interfered with DC maturation. Based on the results that proteinase K and nucleases reduced the effect of LTX-315-treated melanoma supernatant on DC maturation, we concluded that both protein and nucleic acid (DNA and RNA) components in the supernatants of LTX-315-treated melanoma cells contribute to DC maturing.

### Exposure of melanoma cells to LTX-315 results in the release of DC-maturing DAMP/alarmin

Protein quantification by absorbance at the wavelength of 280 nm or BCA assay revealed that the protein levels in the supernatants of LTX-315-treated A375 and B16F10 cells increase in a time-dependent manner (**Supplementary Figures 4A, B**). We next measured whether treatment of A375 or B16F10 melanoma cells with LTX-315 resulted in DAMPS/alarmins release. Western blotting revealed that both high-mobility group box 1 (HMGB1) and high-mobility group nucleosome-binding 1 (HMGN1) began to appear in the culture supernatants within 2 h and reached peak levels at approximately 12 to 18 h after LTX-315 addition (**Figures 2I, J**). This was accompanied by a reduction of HMGB1 and HMGN1 in melanoma cell lysates (**Figures 2I, J**), indicating that LTX-315 treatment results in the translocation/release of both these DAMPs/alarmins from A375 and B16F10 cells into the supernatant. Since both HMGB1 and HMGN1 are known to trigger DC maturation (19–21), these results substantiate the notion that DAMPs/alarmins released by tumor cells in response to LTX-315 treatment contribute to DC maturation.

### LTX-315 form complexes with melanoma cell-derived nucleic acids that promote DC maturation

The data that DNase I and RNase A treatment diminishes DC maturation elicited by the supernatant of LTX-315-treated melanoma cells suggest that nucleic acids (NAs) including DNA and RNA are at least in part responsible for this effect. Necrotic cells are a rich source of extracellular DNA and RNA. Indeed, treatment of A375 and B16 melanoma cells resulted in the release of NAs into the culture supernatants in a time-dependent manner, reaching a concentration of 5 to 10 μg/ml (**Supplementary Figures 5A, B**). Agarose gel electrophoresis revealed that the NAs released in the supernatants by melanoma cells exposed to LTX-315 for 12 h had sizes of approximately 100-300bp (**Supplementary Figures 5C, D**). When these supernatants were observed under a fluorescence microscope after addition of PI, significant number of PI^+^ dots were detected, indictive of the formation of NA aggregates in the supernatants of LTX315-treated A375 and B16 melanoma cells (**Supplementary Figures 5E, F**).

LTX-315 is cationic while NAs are anionic, suggesting the existence of electrostatic interactions. We therefore postulated that the NA aggregates detected in the supernatants of LTX-315-treated melanoma cells might include complexes formed between LTX-315 and DNA/RNA released from necrotic melanoma cells induced by LTX-315. To test this hypothesis, we purified DNA and RNA from the supernatants of LTX-315-treated melanoma (A375 and B16) cells, mixed them with LTX-315, and assessed the formation of complexes. DNA at 10 µg/ml formed larger and larger visible complexes with LTX-315 at concentrations equal to or higher than 50 µg/ml (**Supplementary Figure 6A**). A constant concentration of LTX-315 (20 µg/ml) was chosen to interrogate complex formation with purified DNA/RNA at different concentrations. With increasing concentrations, both DNA and RNA formed increasingly larger complexes with LTX-315 (**Supplementary Figures 6B, C**).

**Figure 3:**
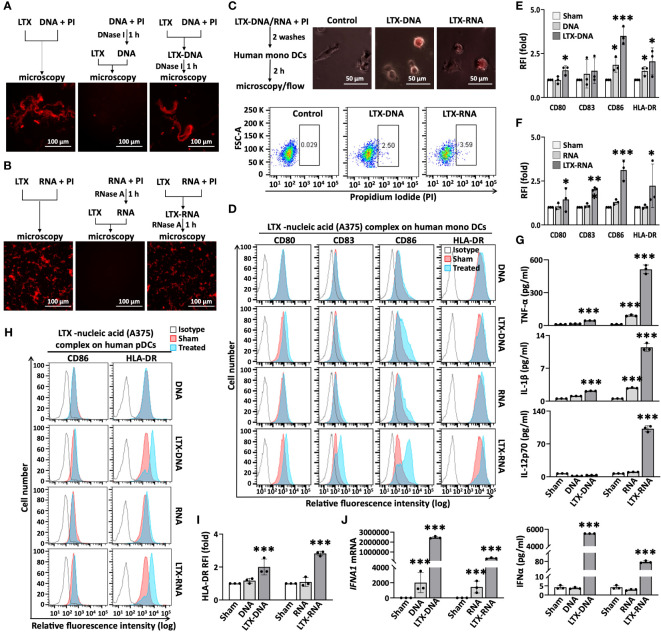
Complexes of LTX-315 and A375-derived nucleic acids (DNA or RNA) induced maturation of monoDCs and pDCs. PI-labeled nucleic acid (NA, 16 µg/ml) and LTX-315 (20 µg/ml) were mixed at 1:1 to allow complex formation (room temperature, 30 min). LTX-315 were added before or after treatment of NA for 1 h with DNase I or RNase **(A)** LTX-315-DNA or LTX-315-RNA complexes protected their NAs from degradation by DNase I **(A)** or RNase A **(B)** as assessed by fluorescence microscopy. LTX-315-NA complexes were engulfed by human monoDCs assessed by fluorescence microscopy and flow cytometry **(C)**. LTX-315-NA complex induction of monoDC **(D–G)** and pDC **(H-J)** maturation. Human monoDCs (5x10^5^/ml) and pDCs (2x10^5^/ml) were treated with LTX-315-NA for 48 and 24 h, respectively before they were analyzed for surface markers and cytokine production. Shown are the plots of one experiment **(D, H)** and the average (mean ± SD) RFI or cytokine concentration **(E, F, G, I, J)** of three independent experiments. *p < 0.05, ***p < 0.001 by Student’s t-test in comparison with the sham.

At least potentially, LTX-315 complexing NAs released by LTX-315-treated cancer cells may allow DNA/RNA to gain access to the endosomal compartment of DCs. Extracellular self-nucleic acids are rapidly degraded by DNases and RNases, which limits their entry into DC endosomes where DNA- and RNA-sensing Toll-like receptor(s) (TLRs) are located. However, self-DNA or self-RNA can become a potent trigger of DC maturation/activation when complexed with endogenous antimicrobial peptides (27–29). To ensure that the formation of complexes between LTX-315 and DNA/RNA indeed prevent the NAs from being degraded, PI-labeled DNA purified from the supernatant of LTX-315-treated A375 cells was treated with DNase I before or after formation of complexes with LTX-315 and subsequently observed by fluorescence microscopy. While LTX-315 and purified DNA efficiently formed complexes (left panel, **Figure 3A**), pretreatment of DNA with DNase I for 1 h before addition of LTX-315 almost completely prevented complex formation due to degradation of DNA (middle panel, **Figure 3A**). In contrast, DNase I added after the formation of LTX-315-DNA complexes could not cause the disappearance of the complexes (right panel, **Figure 3A**). Similarly, addition of RNase A after the formation of LTX-315-RNA complexes also failed to reduce the abundance of these complexes (**Figure 3B**). These results indicated that complexing with LTX-315 protects DNA and RNA from enzymatic degradation.

Next, we explored if LTX-315-DNA and LTX-315-RNA complexes could be engulfed by DCs and cause their maturation. When human monoDCs were incubated with LTX-315-DNA or LTX-315-RNA complexes, they efficiently engulfed them as shown by flow cytometry (**Figure 3C**). Moreover, after co-incubation of monoDCs for 48 h, both LTX-315-DNA and LTX-315-RNA complexes promoted DC maturation as shown by the markedly upregulation of CD80, CD83, CD86, and HLA-DR on DC surface (**Figures 3D-F**) and the production of proinflammatory cytokines such as TNFα, IL-1β, and IL-12p70 (**Figure 3G**). When human peripheral blood pDCs were treated similarly, both LTX-315-DNA and LTX-315-RNA complexes also promoted the expression of CD86 and HLA-DR on DC surface (**Figures 3H, I**), as well as the expression of IFNα at both the mRNA and protein levels (**Figure 3J**). Noticeably, an identical amount of DNA or RNA alone could not induce the activation of either monoDCs or pDCs (**Figures 3D–J**). These results demonstrate that the complexes between LTX-315 and DNA/RNA released from LTX-315-treated melanoma cells have the capacity to induce maturation of human monoDCs and pDCs.

### LTX-315 directly induces the maturation of human monoDCs and pDCs

**Figure 4:**
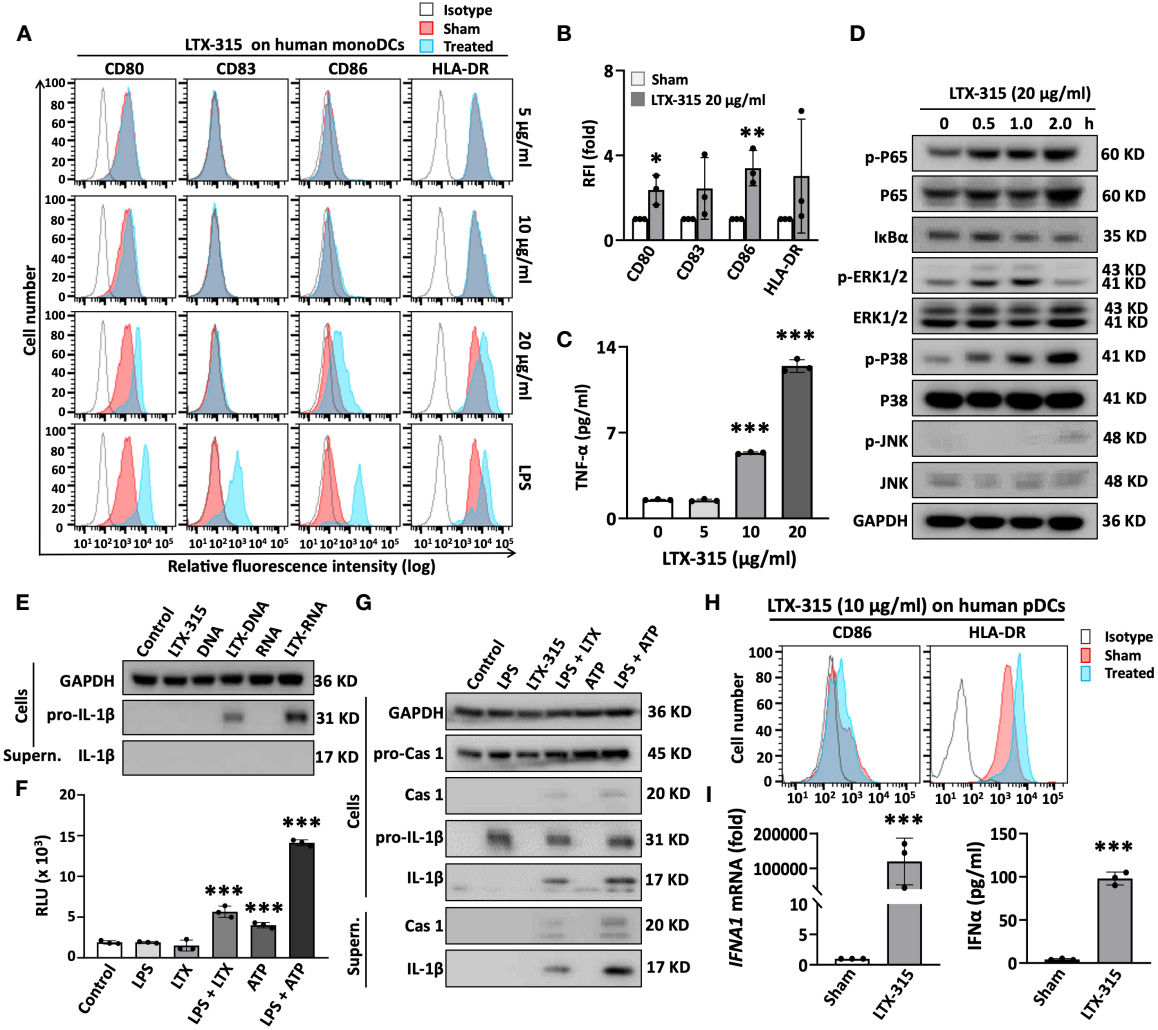
LTX-315 (LTX) directly stimulated the maturation of human monoDCs and pDCs. **(A-C)**, LTX-315 directly induced the maturation of human monoDCs. Human monoDCs incubated at 5 x 10^5^/ml in the absence (sham) or presence of indicated concentration of LTX-315 for 48 h were examined for the expression of surface markers by flow cytometry **(A, B)** and TNFα production by cytokine array **(C)**. **(D)**, LTX-315 triggered NF-κB and MAPK signaling pathways in human monoDCs. DCs treated with LTX-315 as indicated were lysed and the target proteins were detected by Western blot. **(E)**, Complexes of LTX-315-DNA and LTX315-RNA induce the production of pro-IL-1β, but not matured IL-1β, in monoDCs. LTX-315-DNA and LTX-315-RNA complexes were prepared by mixing 25 µl of LTX-315 (20 µg/ml) and 25 µl of DNA or RNA (8 µg/ml) purified from LTX-315-treated A375 cells and incubating at room temperature for 30 min. The mixtures were added to monoDCs and cultured for 8 h before the supernatants (SN) were used for detecting pro-IL-1β and IL-1β by Western blot. **(F, G)**, LTX-315 increased the activity of caspase 1 **(F)** and promoted the cleavage of pro-caspase 1 and pro-IL-1β into caspase 1 and IL-1β **(G)**. monoDCs primed with LPS (100 ng/ml) for 6 h were stimulated with LTX-315 at 20 ng/ml for 5 h or with 5 mM ATP for 1 hour before they were assessed caspase 1 activation **(F)** and IL-1β maturation **(G)**. **(H, I)**, LTX-315 (10 µg) directly induced the maturation of human pDCs **(H)** and promoted the mRNA (left panel) and protein levels (right panel) of IFNα in pDCs **(I)**. Data are shown as the representative data of one experiment **(A, D, E, G, H)** or the average (mean ± SD) of three independent experiments **(B, C, F, I)**. *p < 0.05, **p < 0.01, ***p < 0.001 by Student’s t-test in comparison with the sham or control.

LTX-315 was designed based on the antimicrobial peptide (AMP) lactoferricin (6), and most AMPs including lactoferricin have the capacity to induce DC maturation (30). We therefore investigated if LTX-315 alone could induce maturation of DCs. Treatment of monoDCs with LTX-315 upregulated the surface expression of stimulatory molecules (CD80, CD83 and CD86) and HLA-DR (**Figures 4A, B**), as well as production of TNFα (**Figure 4C**) in a dose-dependent manner, indicating that LTX-315 possesses the capacity to directly induce DC maturation. Noticeably, at this concentration, LTX-315 did not cause DC death (**Supplementary Figures 1C, D**).

DC maturation is always accompanied by the activation of multiple signaling pathways. We next determined if DC maturation-related pathways, NF-κB and major mitogen-activated protein kinases (MAPKs) signaling, were triggered by LTX-315 treatment in monoDCs. LTX-315 treatment of DCs elevated the level of phosphorylation of the p65 subunit of NF-κB and promoted the degradation of I-κBα in time-dependent fashion, without dramatically affecting the level of non-phosphorylated p65 (**Figure 4D**), indicating the activation of NF-κB pathway. LTX-315 also promoted the phosphorylation of ERK1/2, p38, and JNK, with different kinetics, whereas the levels of non-phosphorylated ERK1/2, p38, and JNK appeared to be similar across these samples (indicative of equal loading) (**Figure 4D**). Hence LTX-315 can trigger the activation of NF-κB and multiple MAPK signaling pathways in monoDCs.

The NLR family, pyrin domain-containing 3 (NLRP3) inflammasome also plays an important role in DCs maturation as a source of mature IL-1β. In this setting, activation of DCs to produce IL-1β requires two signals: signal 1 promotes the expression of pro-IL-1β and inflammasome components, and signal 2 activates the NLRP3 inflammasome, resulting in the generation of active caspase 1 (CASP1) that in turn catalyzes the conversion of pro-IL-1β into mature IL-1β. Signal 1 is often provided by pathogen-associated molecular pattern (PAMP)- or DAMP-driven activation of TLRs and NF-κB. Conversely, signal 2 generally emerges from mitochondrial membrane destabilization, K^+^ efflux, reactive oxygen species (ROS) production, and/or lysosomal damage, resulting in the cleavage of pro-caspase 1 into caspase 1. Since LTX-315 has the capacity to permeabilize mitochondrial membrane (8, 9), we speculated that LTX-315 could perhaps also trigger the activation of NLRP3 inflammasome. As determined by immunoblotting, both LTX-315-DNA and LTX-315-RNA complexes, but not LTX-315 alone, promoted the expression of pro-IL-1β in DCs (**Figure 4E**). Next, we treated monoDCs with LPS (signal 1) first and then LTX-315 to determine whether LTX-315 could provide signal 1 in support of inflammasome activation. Measurement of CASP1 activity using a bioluminescent assay revealed that sequential use of LPS and LTX-315 significantly increased CASP1 activity (**Figure 4F**). Similar results were obtained with LPS and ATP or even ATP alone (**Figure 4F**), reflecting the notion that ATP is a potent inflammasome activator. Further immunoblotting analysis of monoDC lysates and supernatants showed that LPS upregulates the expression of pro-CASP and pro-IL-1β in DCs without generating active CASP1 and mature IL-1β (**Figure 4G**). However, treating LPS-primed DCs with LTX-315 generated active CASP1 and coupled with the production of mature IL-1β, as detected in both cell lysates and supernatants (**Figure 4G**). These results demonstrated that LTX-315 acts as signal 2 to trigger the activation of NLRP3 inflammasome in DCs, hence promoting the production of IL-1β.

pDCs are also present in both human and mouse tumor tissues (31). To determine if LTX-315 also activate pDCs, purified human peripheral blood pDCs treated with LTX-315 were analyzed for the upregulation of CD86, HLA-DR and IFNα1. LTX-315 not only dramatically elevated the expression of CD86 and HLA-DR on the pDC surface (**Figure 4H**), but also induced pDC expression of IFNα1 mRNA and secretion of IFNα protein (**Figure 4I**), indicative of a role for LTX-315 in pDC maturation.

### LTX-315-induced maturation of DCs depends on distinct TLRs and MyD88

**Figure 5:**
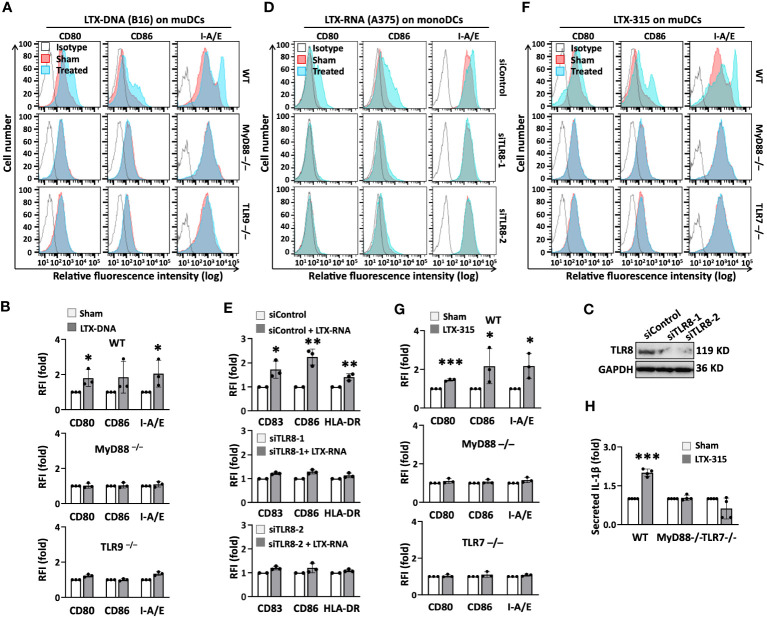
The indirect and direct DC-maturing effect of LTX-315 (LTX) relied on MyD88 and distinct TLRs. **(A, B)**, Complexes of LTX-315 and B16 melanoma cell-derived DNA (ratio of LTX- 315: DNA = 200 ng: 40 ng) upregulation of the expression of CD80, CD86, and I-A/E on mouse DCs was TLR9- and MyD88-depedent. **(C-E)**, Knockdown of TLR8 in human monoDCs abrogated the capacity of complexes of LTX-315 and A375-derived RNA (ratio of LTX-315: RNA = 200 ng: 80 ng) to upregulate DC surface expression of CD80, CD86, and HLA-DR. **(F–H)**, LTX-315 upregulated the expression of surface CD80, CD86, and I-A/E in WT, but not in MyD88-/- or TLR7-/-, DCs **(F, G)**, and DC production of IL-1β **(H)**. DC expression of costimulatory and MHC molecules was measured by flow cytometry **(A, B, D, E, F, G)** whereas the levels of IL-1β in the culture supernatants were quantitated by cytokine array **(H)**. Confirmation of successful TLR8 knockdown was done by Western blot **(C)**. Data are shown as the results of one experiment (A, C, D, F) or the average (mean ± SD) of three independent experiments (B, E, G, H). *p < 0.05, ***p < 0.001 by Student’s t-test compared with the sham.

Our findings suggest that LTX-315 induces DC maturation by both direct and indirect mechanism(s). The capacity of LTX-315-DNA complexes to stimulate the phenotypic maturation (upregulation of CD80, CD86, and I-A/E) of mouse DCs did not occur in DCs derived from TLR9^-/-^ and MyD88^-/-^ mice (**Figures 5A, B**), indicating that the effect of DNA-LTX-315 complex on DC maturation was mediated by TLR9. Since TLR7 and TLR8 have evolved for sensing single-stranded RNA (29), we investigated whether the capacity of LTX-315-RNA complexes to induce DC maturation could be mediated by one of these TLRs. Interestingly, LTX-315-RNA complexes significantly stimulated the maturation of human monoDCs (**Supplementary Figures 7A, B**), but not mouse DCs (**Supplementary Figures 7C, D**), suggesting the involvement of a receptor expressed by humans but not mouse DCs, such as TLR8 (32, 33). To test this possibility, we designed two TLR8-specific siRNAs (siTLR8-1 and siTLR8-2) to knock down TLR8 expression in monoDCs (**Figure 5C**). Of note, siTLR8-1 and siTLR8-2 were equally efficient in abolishing DC maturation induced by LTX-315-RNA complexes (**Figures 5D, E**), establishing that LTX-315-RNA complexes use TLR8 as the receptor to induce human DC maturation.

LTX-315 upregulated the expression of DC surface CD80, CD86, and I-A/E (**Figures 5F, G**) and the production of IL-1β in bone marrow-derived DCs of wild-type (WT) (**Figure 5H**), but not MyD88^-/-^ mice (**Figures 5F, G**), indicating that LTX-315-induced DC maturation relies on the presence of MyD88. Since LTX-315 alone stimulated the maturation of human monoDCs and pDCs (**Figures 4A-I**), as well as mouse bone marrow-derived DCs (**Figure 5F, G**), we next hypothesized that LTX-315 might use a TLR commonly expressed by these cell types. Both TLR7 and TLR9 are expressed by human pDCs (34), immature monoDCs (35) and mouse bone marrow-derived DCs (36). Mouse TLR9^-/-^ DCs responded to LTX-315 similarly as WT DCs, ruling out the potential involvement of TLR9 (**Supplementary Figure 8**). Conversely, LTX-315 failed to upregulate DC expression of CD80, CD86, and I-A/E (**Figures 5A, B**) and secretion of IL-1β (**Figure 5H**) in TLR7^-/-^ DCs. Thus, LTX-315-induced DC maturation is TLR7- and MyD88-dependent.

### MyD88 is required for LTX-315-induced TiDC maturation and homing to dLNs

**Figure 6:**
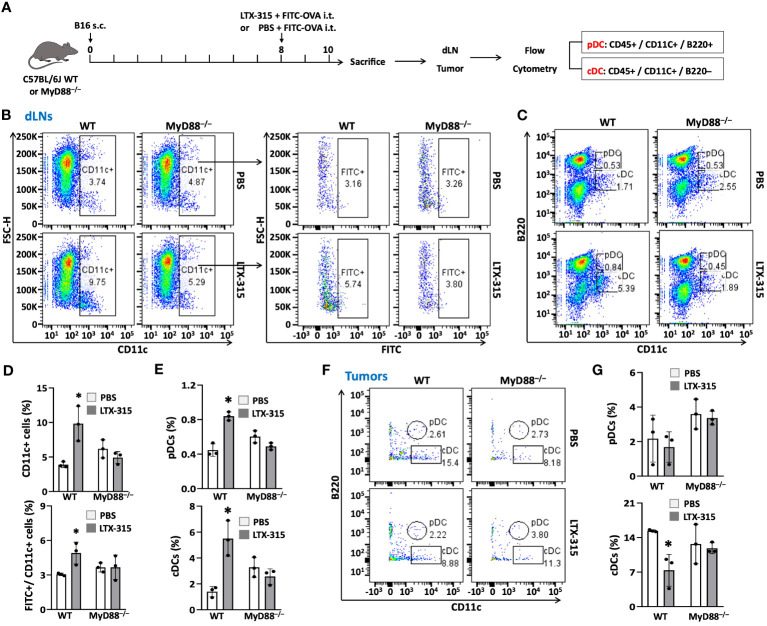
MyD88 knockout abolished the effects of LTX-315 treatment on stimulating TiDC maturation and migration to dLNs. **(A)** Schematic illustration of the flow of the experiment. 1.5 x 10^5^ B16F10 cells were s.c. injected into the right flank of C57BL/6J mice and MyD88^-/-^ mice on day 0. Then mice were i.t. injected 1 µg FITC-OVA mixed with 1 mg LTX-315 or equal volume of 1 µg FITC-OVA in PBS as control on day 8. Tumor tissues and dLNs were collected and analyzed on day 10. **(B-E)**, Percentages of CD11c^+^ DCs and FITC^+^ DCs **(B, C)**, pDCs and cDCs **(D, E)** in dLNs. **(F, G)** Percentages of pDCs and cDCs in tumor tissues. Data are shown as the plots of one mouse **(B, D, F)** or the average (mean ± SD, n=3) percentages **(C, E, G)** of one experiment representative of two. *p < 0.05 by Student’s t test in comparison with the control.

To determine whether MyD88 would also be required for LTX-315 to induce maturation of TiDCs, WT and MyD88^-/-^ mice bearing B16 melanomas were treated with intratumoral LTX-315, using FITC-OVA as a fluorescence tracer to track DC migration from melanoma tissues to dLNs (**Figure 6A**). Consistent with the results of previous experiments (**Figure 1**), intratumoral LTX-315 treatment resulted in the maturation of TiDCs, as evidenced by an increase of CD11c^+^ DCs, FITC^+^ DCs, pDCs, and cDCs in dLNs (**Figures 6B-E**) coupled with a decrease of TiDCs (particularly cDCs) in the melanoma tissues in wild-type mice (**Figures 6F, G**). Importantly, the same did not hold true in MyD88^-/-^ mice (**Figures 6B-G**). These results demonstrate that host MyD88 is required for the ability of LTX-315 to stimulate TiDC maturation homing to dLNs.

### MyD88 is essential for the induction of antitumor immunity by LTX-315 treatment

**Figure 7:**
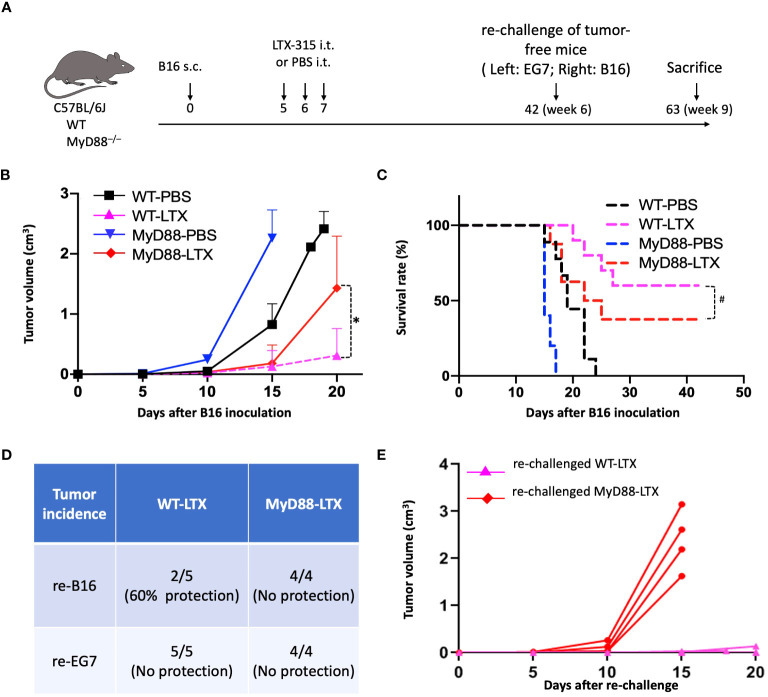
MyD88 knockout compromised the generation of anti-B16 immunity in response to LTX-315 (LTX) treatment. **(A)** Schedule of the experiment. C57BL/6J mice and MyD88^-/-^ mice (female, 8 wks, n = 8-10) were s.c. inoculated into the right flank with 1.5 x 10^5^ B16F10 cells on day 0. Mice were i.t. treated with 3 consecutive daily injection of 1 mg LTX-315 or equal volume of PBS. Tumor-free mice were s.c. re-challenged with 1.5 x 10^5^ B16F10 and 4 x 10^5^ EG7 cells into the right flank and left flank, respectively. **(B, C)**, B16 tumor growth **(B)** and survival **(C)**. **(D, E)**, Incidence of EG7 and B16 tumors on Day 15 after rechallenge **(D)** and growth of re-challenged B16 tumors in LTX-315-cured WT and MyD88-/- **(E)** mice. *p < 0.05 and #p < 0.05 by Repeated Measures Analysis of Variance (ANOVA) and Log-rank test, respectively.

To examine whether MyD88 mediates the ability of LTX-315 to elicit adaptive anticancer immunogenity, WT C57BL/6J and congenic MyD88^-/-^ mice were inoculated with B16F10 tumors and treated with an i.t. injection of either PBS (as vehicle control) or LTX-315 (1 mg/injection/day) for 3 consecutive days beginning on day 5 post B16F10 inoculation (**Figure 7A**). Consistent with a previous report (11), LTX-315 strongly inhibited tumor growth in WT mice (**Figure 7B**), an effect that was partially lost in MyD88^-/-^ mice (**Figure 7B**), suggesting that MyD88 is involved in the therapeutic effects of LTX-315. In line with these findings, LTX-315 treatment provided B16F10-bearing mice with an improved overall survival extension compared to B16-bearing MyD88^-/-^ mice (**Figure 7C**). Finally, cured tumor-free mice were re-challenged with B16F10 and unrelated EG7 cells on contralateral flanks, followed by monitoring the formation and growth of re-challenged tumors to assess the generation of tumor-specific immunity. In this setting, all mice developed progressing EG7 tumors (**Figure 7D**). Conversely, out of 5 re-challenged WT mice, 60% (3/5) were protected from the establishment of B16F10 tumors, while 40% (2/5) had small slow-growing B16F10 lesions, indicating that the generation B16F10-specific immunity and long-term memory (**Figure 7D**). Importantly, none of the 4 MyD88^-/-^ mice cured of primary B16F10 tumors by LTX-315 treatment exhibited any form of protection against re-challenge with B16F10 cells (**Figures 7D, E**). These results provide *in vivo* evidence to support the essential role of MyD88 in the ability of LTX-315 to induce protective anti-melanoma immunity upon intratumoral administration.

## Discussion

LTX-315 is a cationic oncolytic peptide that upon intratumoral administration can stimulate the generation of potent anticancer immune responses by promoting necrotic cancer cell death (5, 6). In this setting, lysed cancer cells are likely taken up by APCs (mainly TiDCs), allowing processing and presentation of tumor antigen(s) in the context of MHC class I and II molecules. Thus, once antigen-loaded DCs migrate to dLNs, they can present tumor antigens to T cells, resulting in generation of either immunity or immune tolerance (18, 37), depending on whether TiDCs are properly matured to express high levels of co-stimulatory molecules (e.g. CD80, CD83, CD86) and immunostimulatory cytokines (37). Despite abundant evidence on the immunotherapeutic effects of LTX-315 (38, 39), whether and how LTX-315 treatment results in the maturation of TiDCs remained to be elucidated. Our findings demonstrate that the MyD88-dependent maturation of TiDCs (including cDCs and pDCs) coupled with their migration to dLNs, plays a central role in the induction of tumor-specific immunity as elicited by the intratumoral administration of LTX-315.

One of the important findings of the current study is the demonstration that intratumoral injection of LTX-315 results in TiDC maturation. TiDCs (especially cDCs) are particularly adept at initiating T cell responses against malignant lesions and directing T cell polarization in support of cytotoxic Th1 polarized immunity (34). pDCs are the major producer of type I IFNs and can also act as APCs for both CD4^+^ and CD8^+^ T cells (40, 41). Owing to selective expression of TLR7 and TLR9 (34), pDCs can detect unmethylated CpG-rich DNA motifs or single strand RNA (42). Therefore, the maturation and migration of both cDCs and pDCs from tumors to corresponding dLNs in response to LTX-315 treatment suggest that both types of TiDCs are presumably involved in the induction of LTX-315-driven antimelanoma immunity. Additional work is required to confirm this possibility. Moreover, whether the immunotherapeutic activity of LTX-315 in other tumor models also involve both cDCs and pDCs remains to be investigated.

The present study indicates that three distinct pathways are likely to underlie the ability of LTX-315 to induce DC maturation. First, LTX-315 treatment of cancer cells results in the release of DAMP/alarim HMGB1 (7, 8, 14) and HMGN1 (present study). Both HMGB1 and HMGN1 utilize the TLR4-mediated signaling as the predominant pathway for inducing DC maturation and induction of immune responses including antitumor immunity (20, 30, 43, 44). This pathway of LTX-315-induced TiDC maturation is at least in part dependent on the signal transducer MyD88.

The second pathway by which LTX-315 induces DC maturation involves the formation of complexes between LTX-315 and NAs (DNA and RNA) released from LTX-315-treated cancer cells. Generally, endogenous self-DNA and self-RNA are not immunostimulatory due to rapid degradation by DNase and RNase and a failure to access endosomal TLRs (28). However, self-DNA and self-RNA can trigger DC activation if they are complexed with alarmins/DAMPs such as defensins or cathelicidins and engulfed by DCs (27, 28). LTX-315 not only induces the release of DNA and RNA from dying cancer cells, but also form complexes with them. Such complexes are protected from degradation and hence can gain easier access to DC endosomes for improved opportunity to stimulate the maturation of DCs that expressed both TLR8 and TLR9 (35, 40, 41, 45).

The third pathway involves direct stimulation of DC maturation by LTX-315. LTX-315 directly activates multiple intracellular signaling cascades, including NF-κB, multiple MAPKs, and NLRP3 inflammasome, to promote upregulation of DC surface costimulatory and MHC molecules and DC production of TNFα and IL-1β. This direct effect of LTX-315 on DC maturation is by mediated by TLR7. The precise mechanism by which LTX-315 peptide activates TLR7 remains to be elucidated, but the chemical entities of the presence of indole ring structure in the tryptophan residues and the amphipathic nature of LTX-315 may contribute, since similar chemical structures are known to activate TLR7.

The three pathways by which LTX-315 treatment induces DC maturation converge on the signal transducer MyD88. In line with this notion, LTX-315-induced maturation and dLN homing of TiDCs was diminished in B16 melanoma-bearing MyD88-/- mice. Similarly, host MyD88 was absolutely required for LTX-315 to induce the establishment of long-term immunological memory downstream of tumor eradication. These data suggest that cancer patients bearing hypofunctional MyD88 variants or expressing limited levels of MyD88 may exhibit reduced responses to LTX-315. Such a possibility remains to be formally interrogated in clinical settings.

Despite this and other unknowns, our data cast additional light on the signaling pathways through which LTX-315 engages adaptive anticancer immune responses, i.e., a multipronged mechanism involving both direct and indirect triggering of multiple TLRs that converge on MyD88 signaling in DCs. It will be important to understand the best approaches to harness this mechanism to improve the clinical activity of LTX-315.

## Data availability statement

The original contributions presented in the study are included in the article/**Supplementary Material**. Further inquiries can be directed to the corresponding author.

## Ethics statement

The animal study was approved by NCI Animal Care and Use Committee. The study was conducted in accordance with the local legislation and institutional requirements.

## Author contributions

XL: Conceptualization, Data curation, Formal Analysis, Investigation, Methodology, Validation, Writing – original draft. TY: Data curation, Formal Analysis, Validation, Writing – review & editing. TH: Investigation, Methodology, Writing – review & editing. MA: Investigation, Methodology, Writing – review & editing. JL: Investigation, Methodology, Writing – review & editing. AT: Investigation, Project administration, Writing – review & editing. BS: Funding acquisition, Resources, Writing – review & editing. ØR: Resources, Writing – review & editing. LG: Formal Analysis, Funding acquisition, Writing – review & editing. JO: Conceptualization, Funding acquisition, Writing – review & editing. **DY:** Conceptualization, Data curation, Formal Analysis, Investigation, Methodology, Supervision, Writing – original draft, Writing – review & editing.
