# Supplementary material for: LTX-315 triggers anticancer immunity by inducing MyD88-dependent maturation of dendritic cells
Source: Front Immunol. 2024 Mar 13;15:1332922. doi: 10.3389/fimmu.2024.1332922 (PMC10967226; doi:10.3389/fimmu.2024.1332922)
Supplement: Supplementary file 1 [file DataSheet_1.docx]

**Supplementary Figures**


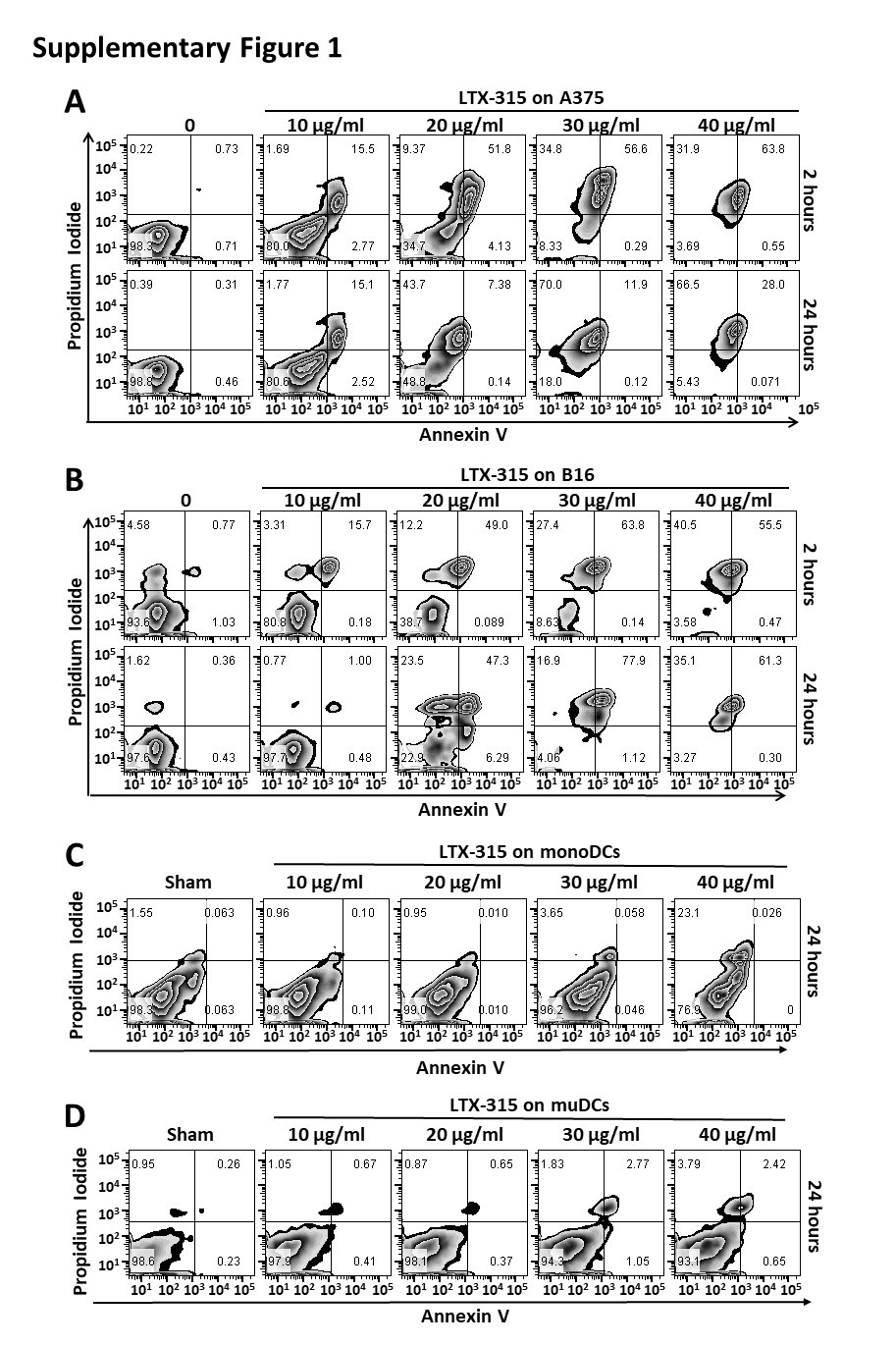


**Supplementary Figure 1. LTX-315 induced necrotic death of melanoma cells**. Cell morphology change of LTX- 315-treated human A375 (**A**), mouse melanoma B16F10 (**B**) cells, human monocyte DCs (monoDCs; **C**) and mouse DCs (muDCs; **D**) at different time points with various concentrations. Flow cytometry analysis of cells treated with LTX-315 at the times and concentrations as indicated after staining annexin V-FITC and PI.


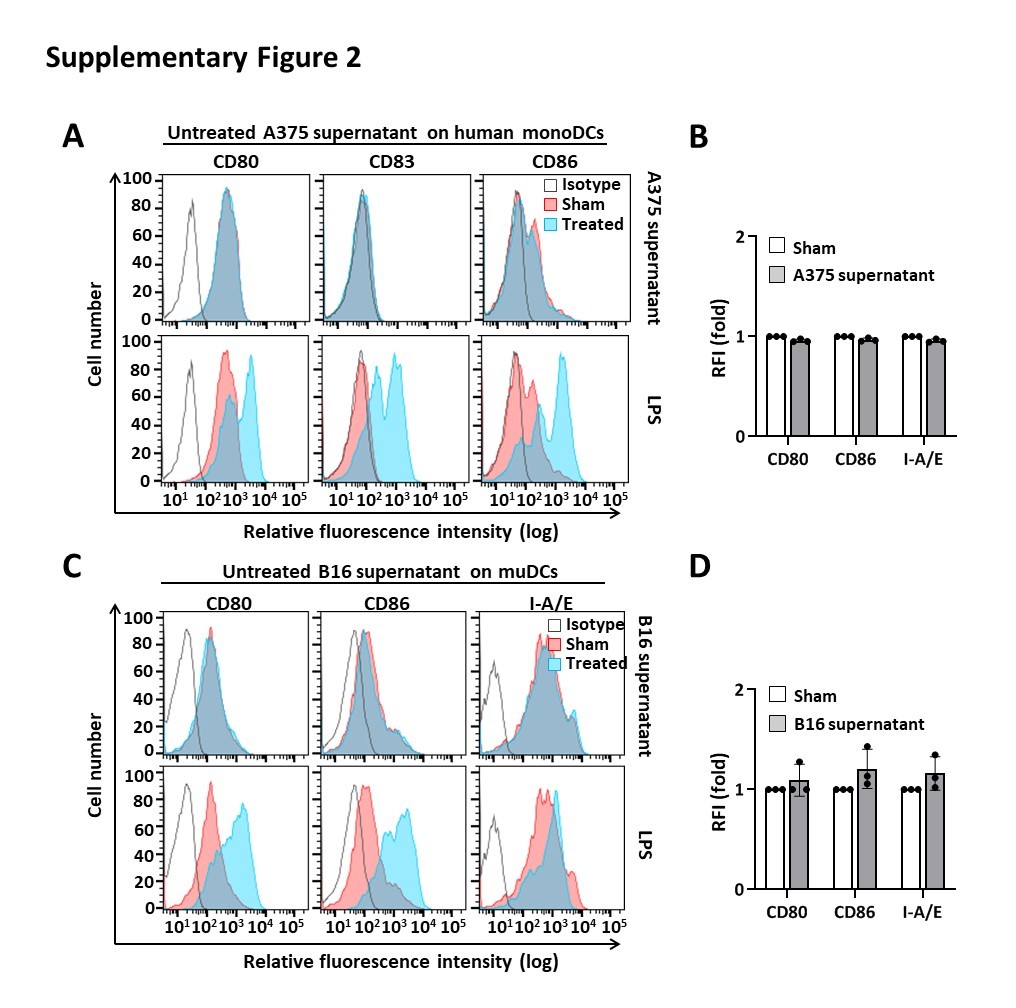


**Supplementary Figure 2. The culture supernatants of melanoma cells did not stimulate the maturation of DCs.** monoDCs and muDCs were treated with 80% of A375 and B16F10 supernatant, respectively for 48 h before they were assessed for the expression of costimulatory and MHC molecules by flow cytometry. Shown are the histograms of one experiment (**A, C**) and the average (mean ± SD, n=3) of relative fluorescent intensity (RFI) in terms of fold change (**B, D**).


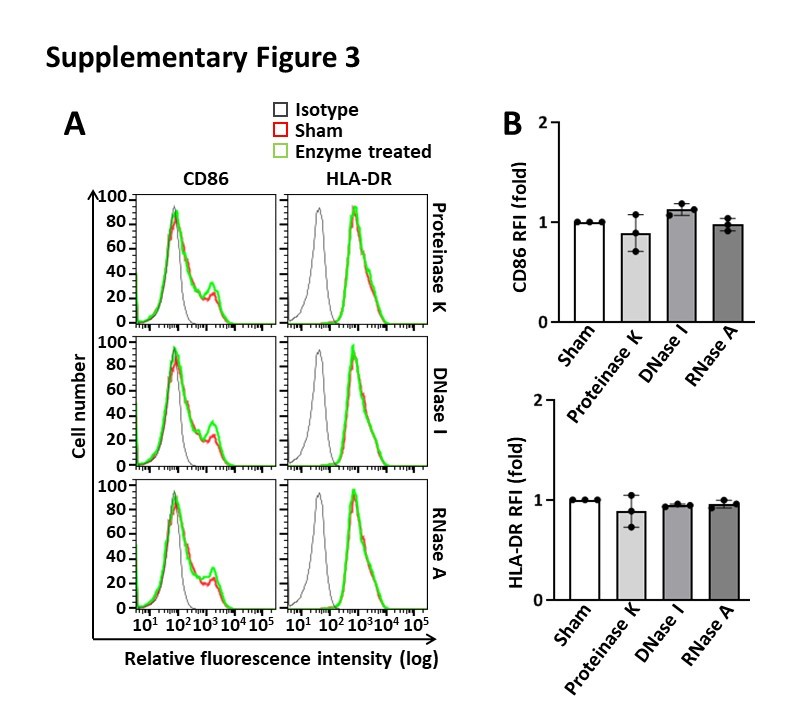


**Supplementary Figure 3. Treatment of monoDCs with proteinase K, DNase I, or RNase A did not affect their maturational status.** Shown are the histograms of one experiment (**A**) and the average (mean ± SD) of relative fluorescent intensity (RFI) in terms of fold change of three experiments (**B**).


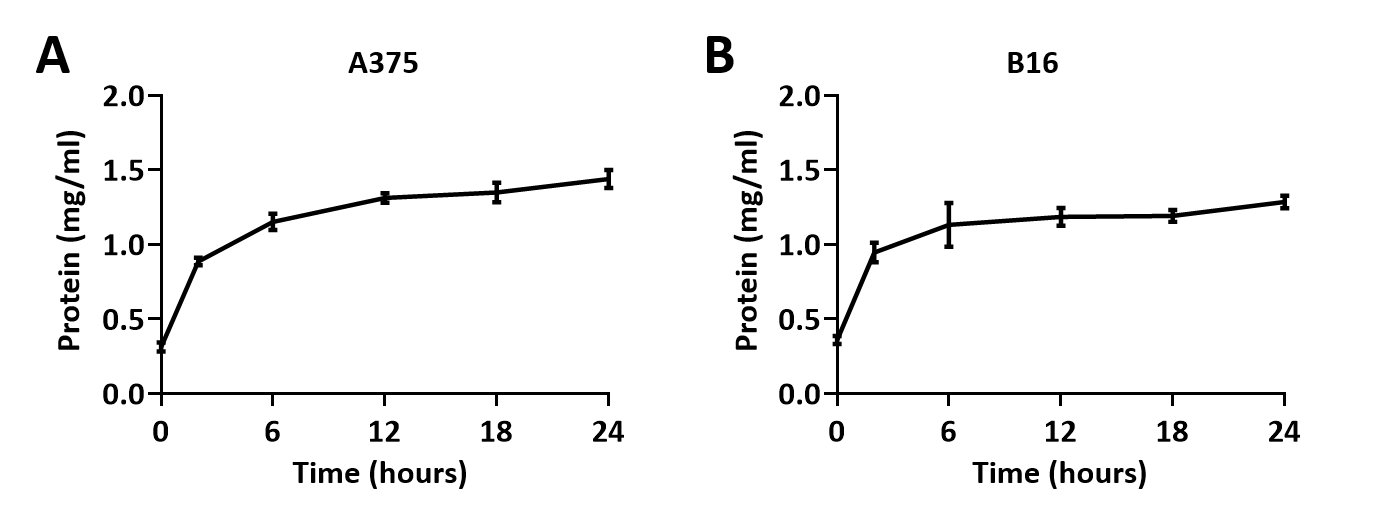


**Supplementary Figure 4. LTX-315 treatment of melanoma cells resulted in rapid release of proteins.** A375 and B16F10 melanoma cells were treated with 50 μg/ml LTX-315 in FBS-free RPMI 1640 medium for indicated time. Proteins released into the supernatant of LTX-315-treated A375 (**A**) or B16F10 (**B**) cells were quantitated by BCA assay. The data are presented as means ± SD from three independent experiments.


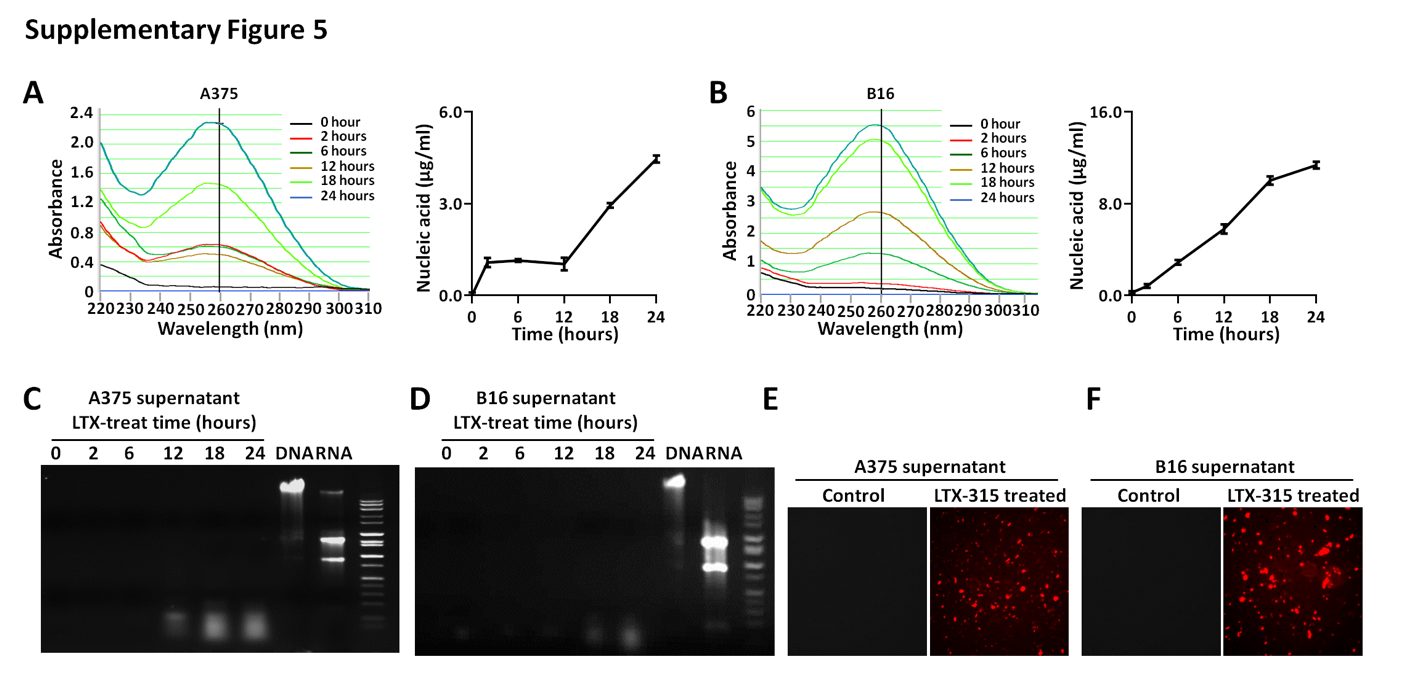


**Supplementary Figure 5. LTX-315 treatment of melanoma cells resulted in release of nucleic acids (DNA and** **RNA) that formed complexes with with LTX-315. A-D**, NAs were released from LTX-315-treated A375 (**A, C**) and B16F10 (**B, D**) into the supernatant in a time-dependent manner. Soluble NA fragments (~100-300bp) were detected in the supernatant of A375 (**C**) and B16F10 (**D**) after 12 h treatment. **E-F**, PI+ NA aggregates were observed in the supernatant of A375 (**E**) and B16F10 (**F**) cells at 6 h after the addition of LTX-315. The data are presented as means ± SD from three independent experiments.

**
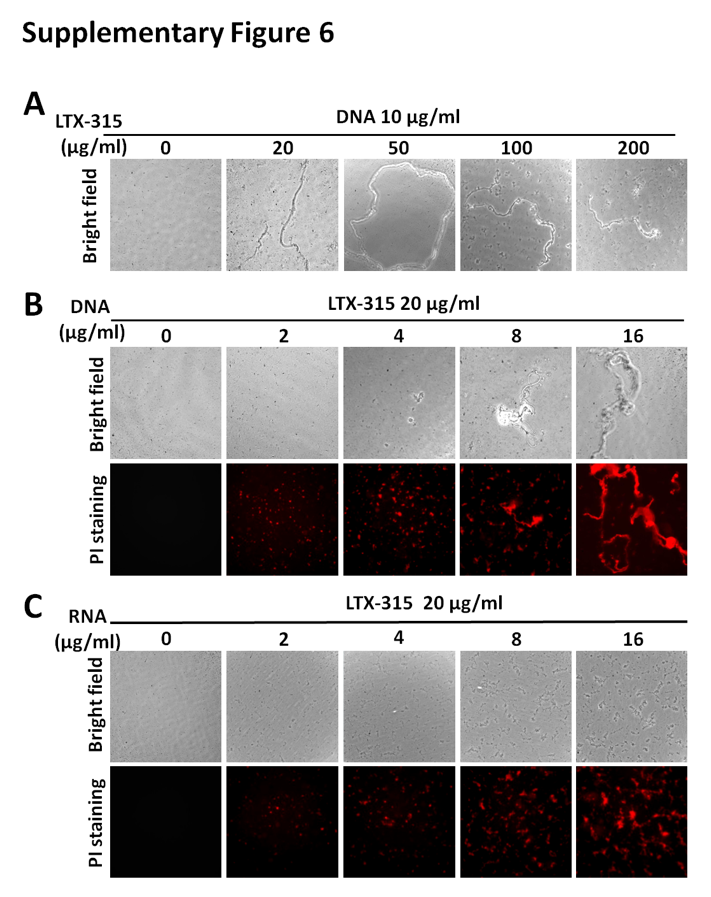
**

**Supplementary Figure 6. LTX-315 formed complexes with DNA and RNA purified from the supernatants of melanoma cells treated with LTX-315. A,** Complexes formed between DNA (10 μg/ml) and indicated concentrations of LTX-315. **B-C**, Complexes formed between LTX-315 (20 μg/ml) and various concentrations of PI-labeled DNA (**B**) and RNA(**C**).


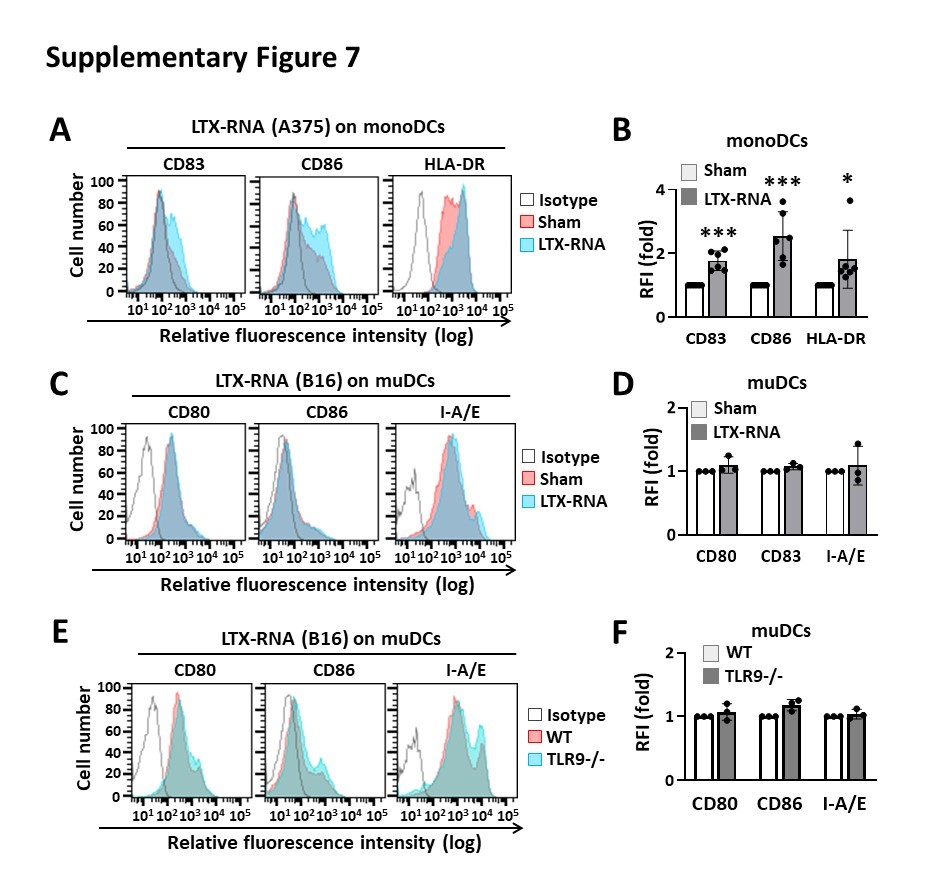


**Supplementary Figure 7. LTX-315-RNA complexes induced the maturation of human but not mouse DCs.** The LTX-315-RNA complexes was formed by mixing LTX-315 (20 µg) and RNA (8 µg) and incubating at room temperature for 30 min. Human (**A,B**) or mouse (**C,D**) DCs treated with the LTX-315-RNA complexes were analyzed by flow cytometry. Shown are the representative plots of one experiment (**A,C**) or average (mean± SD) RIF of three independent experiments in terms of fold change (**B,D**). **p* < 0.05, ****p* < 0.001 by Student’s t-test in comparison with the sham.


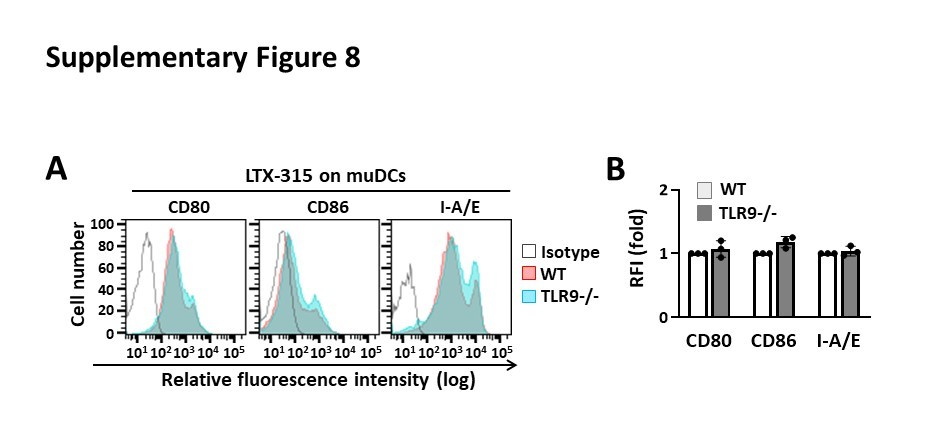


**Supplementary Figure 8. TLR9 knockout did not abolish the direct effect of LTX-315 on muDCs.** muDCs isolated from wildtype (WT) and TLR9^-/-^ C57BL/6J mice were treated with LTX-315 (20 µg) for 48 hours and analyzed by flow cytometry. Shown are the representative plots of one experiment (**A**) or average (mean± SD) RIF of three independent experiments in terms of fold change (**B**).
